# mRNA in cancer immunotherapy: beyond a source of antigen

**DOI:** 10.1186/s12943-021-01329-3

**Published:** 2021-03-03

**Authors:** Lien Van Hoecke, Rein Verbeke, Heleen Dewitte, Ine Lentacker, Karim Vermaelen, Karine Breckpot, Sandra Van Lint

**Affiliations:** 1 VIB-UGent Center for Inflammation Research, Technologiepark 71, 9052 Ghent, Belgium; 2 Department of Biomedical Molecular Biology, Ghent University, Technologiepark 71, 9052 Ghent, Belgium; 3 Ghent Research Group on Nanomedicines, Lab for General Biochemistry and Physical Pharmacy, Department of Pharmaceutical Sciences, Ghent University, Ottergemsesteenweg 460, 9000 Ghent, Belgium; 4 Cancer Research Institute Ghent (CRIG), Ghent University, Ghent, Belgium; 5 Tumor Immunology Laboratory, Department of Respiratory Medicine and Immuno-Oncology Network Ghent, Ghent University Hospital, Corneel Heymanslaan 10 MRB2, 9000 Ghent, Belgium; 6 Laboratory for Molecular and Cellular Therapy, Department of Biomedical Sciences, Vrije Universiteit Brussel, Laarbeeklaan 103 Building E, 1090 Brussels, Belgium

**Keywords:** Cancer, mRNA, Nanoparticle, Antibody, Dendritic cell, T cell, Cytokine, Tumor microenvironment

## Abstract

mRNA therapeutics have become the focus of molecular medicine research. Various mRNA applications have reached major milestones at high speed in the immuno-oncology field. This can be attributed to the knowledge that mRNA is one of nature’s core building blocks carrying important information and can be considered as a powerful vector for delivery of therapeutic proteins to the patient.

For a long time, the major focus in the use of in vitro transcribed mRNA was on development of cancer vaccines, using mRNA encoding tumor antigens to modify dendritic cells ex vivo. However, the versatility of mRNA and its many advantages have paved the path beyond this application. In addition, due to smart design of both the structural properties of the mRNA molecule as well as pharmaceutical formulations that improve its in vivo stability and selective targeting, the therapeutic potential of mRNA can be considered as endless.

As a consequence, many novel immunotherapeutic strategies focus on the use of mRNA beyond its use as the source of tumor antigens. This review aims to summarize the state-of-the-art on these applications and to provide a rationale for their clinical application.

## Background

Cancer is an umbrella term for life-threatening diseases that are characterized by an uncontrolled growth of transformed, malignant cells that, when left untreated, can disseminate throughout the body. Treating cancer is challenging, as conventional therapies, such as surgery, radiotherapy and chemotherapy, although improved, are unable to prevent disease recurrence in a significant number of patients [1].

A better understanding of the tumor and its environment, in particular the knowledge that various immune cells, such as natural killer (NK) cells and CD8^+^ T cells, can infiltrate tumors and can act in concert to kill tumor cells, has fueled the development of novel cancer therapy strategies that aspire activation of the immune system. Examples thereof are cancer vaccination, adoptive cell therapy, the use of cancer-specific monoclonal antibodies (mAbs) as well as modulation of the immunosuppressive tumor microenvironment (TME) with blockade of inhibitory immune checkpoint pathways being extensively studied [2–6]. These strategies, either alone or combined with conventional therapies, have changed how cancer is being treated anno 2020.

The use of in vitro transcribed (IVT) mRNA for development of cancer therapeutics has received growing interest. This is attributed to the knowledge that mRNA is a powerful vector for delivery of therapeutic proteins to the patient, which can be further potentiated through modifications to the mRNA molecule as well as its formulation in smartly designed nanoparticles [7]. In this regard, the use of IVT mRNA encoding tumor antigens has been extensively studied for the development of potent cancer vaccines. The different steps in the development of these mRNA-based vaccines have been extensively described elsewhere [8, 9]. mRNA has also been studied as a means to (1) deliver cancer-specific and immune checkpoint blocking mAbs, (2) modulate the tumor site itself to promote tumor cell killing and/or inhibit immunosuppression or (3) generate tumor-specific T cells. These non-tumor antigen mRNA-based immunotherapy approaches have proven successful in preclinical studies, which have encouraged their evaluation in a clinical setting.

In this review, we provide a concise, yet comprehensive overview of the current state-of-the-art on the use of mRNA as a vector for in situ delivery of therapeutic proteins beyond tumor antigens. These include mAbs or antibody fragments, cytokines and proteins with immune activating potential. As an exception to the in situ delivery of mRNA encoding therapeutic proteins, we also introduce the use of mRNA for the therapeutic engineering of tumor-specific T cells in the context of adoptive cell therapy. Because the efficacy of mRNA-delivered therapeutic proteins largely depends on the level, persistence and location of expression, we start this review with discussing the requirements of IVT mRNA for these approaches and strategies to efficiently deliver the mRNA to the desired cell type and/or organ.

### From structure to pharmacology: engineering mRNA molecules for in vivo applications

#### Structural properties of IVT mRNA

IVT mRNA is synthesized in cell-free circumstances from a linearized DNA template using highly efficient and promotor-specific bacteriophage-derived RNA polymerases (SP6, T7 or T3) [10]. Fully processed mRNA consists of five in *cis*-acting structural elements. From 5′ to 3′ end these include: (1) a cap structure; (2) a 5′ untranslated region (UTR); (3) the coding sequence of the desired therapeutic protein; (4) a 3′ UTR and (5) a sequence of repeated adenine nucleotides forming a poly(A) tail. Technological advancements in mRNA engineering, including the emergence of multiple variants of these individual structural elements, and their impact on mRNA stability and translation efficiency are the subject of many reviews [11–15].

Over the last decades, a better understanding of mRNA’s structural biology has led to the production of mRNA constructs with increasingly improved stability and translation capacity. A critical determinant here is the recognition of mRNA molecules by innate immune sensors. Upon cell entry mRNA molecules are detected by pattern recognition receptors (PRRs), including endosomal Toll-like receptors (TLR3 [16] and TLR7/8 [17]) and cytosolic nucleic acid sensors (MDA-5 and RIG-1) [18], which results in cytokine signaling and the induction of anti-viral immunity. While the innate immune activity of mRNA can be of critical importance for the potency of mRNA-based cancer vaccines, immunostimulatory effects should be minimized for the mRNA-based approaches discussed in this review [19]. Indeed, the onset of programmed anti-viral mechanisms can drastically lower the mRNA half-life and translation efficiency, while it is also a potential driver of adverse effects [20, 21].

**Fig. 1:**
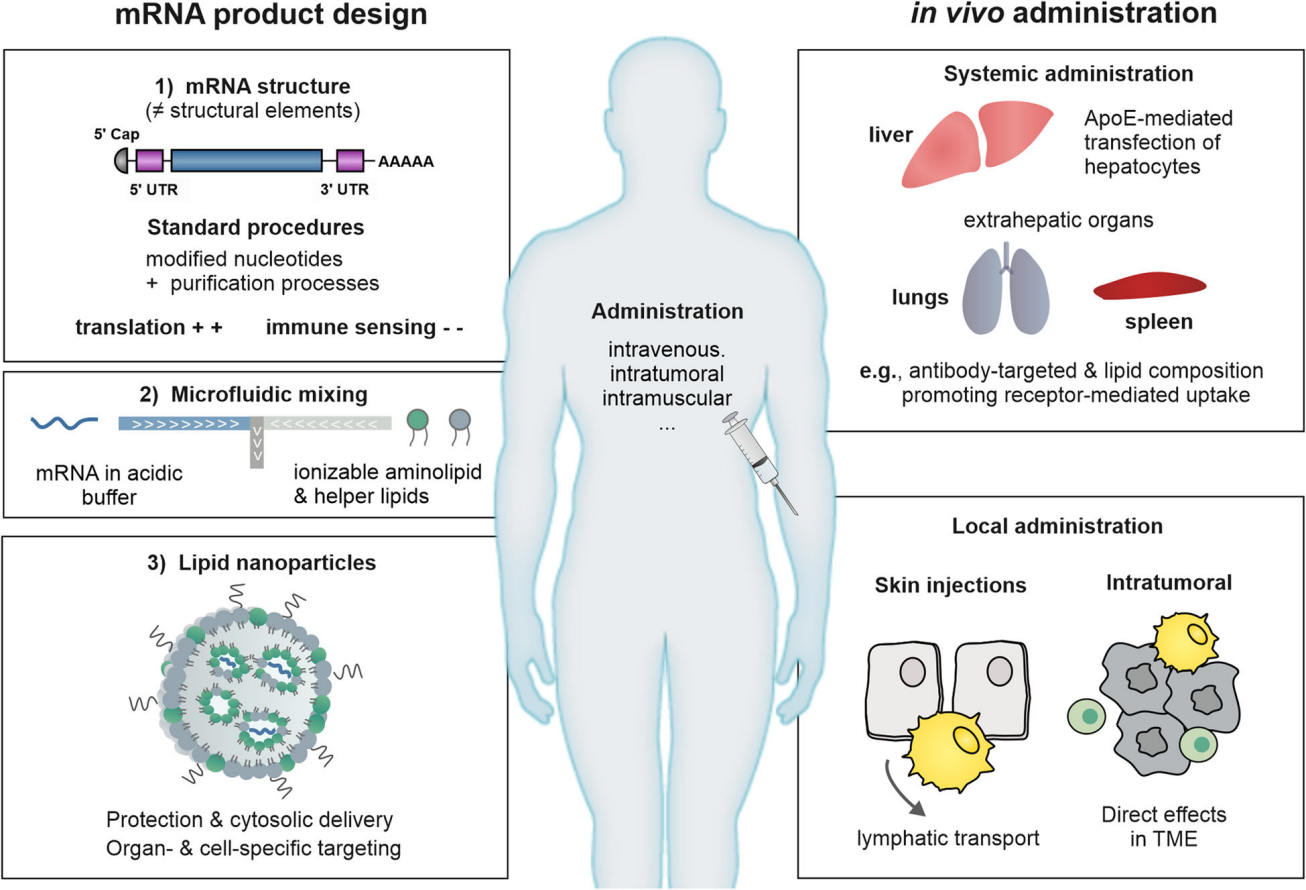
Design and in vivo administration of mRNA therapeutics. Left box: mRNA product design. mRNA consists of five in *cis*-acting structural elements: (1) a cap structure; (2) a 5′ untranslated region (UTR); (3) the coding sequence of the desired therapeutic protein; (4) a 3′ UTR and (5) a sequence of repeated adenine nucleotides forming a poly(A) tail. By using modified nucleotides and extensive purification processes, the innate immune stimulation characteristic of mRNA can be down-modulated. Nowadays, lipid-based nanoparticles (LNPs) are often used for the delivery of mRNA therapeutics by making use of microfluidic mixing devices. In such devices, an acidic aqueous solution of mRNA is rapidly mixed with an ethanol solution of lipids. By using LNPs, the mRNA is protected from degradation and is delivered to the cytosol. Optionally, specific targeting entities can be added to the surface of LNPs to direct the mRNA-LNP to specific tissues or cells. Right box: in vivo administration of the mRNA therapeutic. The systemic delivery of mRNA complexed in LNPs mainly target the liver due to the binding of apolipoprotein E and subsequent receptor-mediated uptake by hepatocytes. To circumvent predominant hepatic uptake and expression, the lipid composition of the LNP can be adjusted or specific targeting Abs can be added to the LNPs. Furthermore, upon local delivery by intradermal, intramuscular or subcutaneous administration of mRNA-LNPs, mainly expression at the site of injection has been observed. Abbreviations: UTR: untranslated region; LNP: lipid-based nanoparticles; TME: tumor microenvironment

A major breakthrough in this regard, was the discovery that post-translational modifications to the mRNA nucleotides in mammalian cells prevent the immune recognition of mRNA [22]. As an outstanding example, the incorporation of N1-methyl-pseudouridine (1mΨ) in mRNA was found to alter the interaction between mRNA and PRRs (in particular TLR3, TLR7 and RIG-I), thereby down-modulating the innate immune stimulation by mRNA [23–25]. Moreover, a study by Mauger et al. [26, 27] evidenced that the replacement of uridine with 1mΨ stabilizes secondary structures in mRNA molecules, where they identified that a flexible leader region and a high degree of secondary structure throughout the remainder of the molecule correlated with a prolonged functional half-life of mRNA and higher protein expression. Supported by findings of several studies [28, 29], the authors suggested that incorporation of modified uridine not only helps mRNA to evade innate immunity but might also affect the binding and dynamics of mRNA molecules with ribosomes. In addition, double stranded RNA (dsRNA) impurities formed during transcription significantly contribute to the innate immune activity of mRNA therapeutics [30]. Purification of IVT mRNA by means of high-performance liquid chromatography (HPLC), or alternative simplified methods using cellulose columns or selective digestion of dsRNA fragments using RNAse III have been proposed [30–32]. In an elegant study, Nelson and colleagues recently demonstrated that by combining 1mΨ-modified mRNA with the removal of dsRNA impurities through process optimization and/or purification, mRNA products can be obtained with an almost completely immunologically silent and most favorable expression level [24]. Figure 1 gives an overview of the structural properties and design of mRNA therapeutics.

Thess and colleagues showed in 2015 that sequence-optimized, HPLC-purified, unmodified mRNA can also achieve sufficient protein expression and avoid immunogenicity [33]. In their study, the codons of the open reading frame (ORF) are adapted in order to improve translation and half-life of the mRNA. To this end, only the most GC-rich codons were used for each amino acid. Moreover, various biological sources were screened to identify potent enhancer and stabilizer elements. In this way an optimized ORF sequence is accompanied with an optimal combination of untranslated sequences. Remarkably, unmodified sequence-engineered mRNA constructs could be as competitive as modified mRNA molecules in large primates [33]. This is in contrast with the reports of Karikó showing that incorporation of modified nucleotides leads to superior non-immunogenic mRNA with increased translation and stability [30, 34, 35]. A possible explanation for this discrepancy arises from variations in RNA sequence optimization, the stringency of mRNA purification to remove dsRNA contaminants and the level of innate immune sensing in the targeted cell types.

#### Delivery systems – the success of mRNA lipid nanoparticles

By formulating mRNA in nanoparticles, the mRNA can be protected from harsh biological conditions, and cellular uptake and release of the mRNA from endosomes can be maximized, while the use of additional targeting moieties and/or tuning of particle characteristics can potentially promote organ- and cell-specific targeting. Today’s most clinically advanced mRNA therapeutics make use of lipid-based nanoparticles (LNPs). These LNPs are typically manufactured in a single-step procedure using microfluidic mixing devices, which enables straightforward (up) scaling and translation from bench to GMP. In these devices, an acidic aqueous solution of mRNA is rapidly mixed with an ethanol solution of lipids. By diluting the ethanol phase, the lipids undergo a condensation process and spontaneously form lipid vesicles while entrapping the mRNA. Standard components of such LNP delivery system are (1) a cationic lipid, which complexes the negatively charged RNA and facilitates cytosolic delivery, in combination with other structural “helper” lipids such as (2) cholesterol, (3) phospholipids and (4) a diffusible polyethylene glycol (PEG)-conjugated lipid.

A critical step in the optimization of LNPs, initially for the delivery of small interfering RNA (siRNA), was the introduction of a new class of “ionizable” cationic lipids. Most prominent examples are LNPs composed with D-Lin-MC3-DMA or the lipidoid C12–200 [36–38]. The apparent pKa value of these ionizable lipids (pKa < 7) makes these lipids positively charged during the RNA complexation (at low *p*H), but LNPs remain relatively neutral at physiological *p*H. In comparison to formulations previously used for gene delivery, ionizable LNPs were found to be much more powerful to transfect liver cells, while charge-related toxicities could be minimized. A study by Akinc et al. [39] demonstrated that this could be attributed to the absorption of apolipoprotein E and enhanced receptor-mediated clearance by hepatocytes via the low-density lipoprotein receptor. Follow-up studies demonstrated that by optimally adjusting parameters such as the molar ratios of lipids, the lipid-to-RNA ratio, and/or type of phospholipid in the particle, liver targeting could be further improved which eventually revolutionized the field with the approval of Patisiran as a first-of-its-kind siRNA therapeutic in humans [40].

Over time, the use of ionizable LNPs became commonplace for the in vivo delivery of mRNA [12, 41]. Recently, advances were made in discovery of new lead ionizable lipids with enhanced bio-degradability, new combinations with alternative helper lipids, such as cholesterol analogues and polysarcosine, as an alternative surface modification to PEG [42–45]. When administered systemically, LNPs provide an excellent approach for direct delivery of mRNA therapeutics to liver cancers, or to exploit the liver as a factory site for the systemic secretion of mRNA-encoded proteins [46, 47]. Moreover, LNP formulations can be designed to also target organ (cancer) tissues beyond the liver, maybe even to achieve cell-specific delivery. Early reports showed that LNP design can be optimized for the targeting of inflammatory monocytes and antigen-presenting cells (APCs) in spleen and bone marrow [48, 49]. Other studies have focused on the use of antibody conjugates to promote receptor-mediated uptake by specific target cells, and as a means to re-target LNPs to the lungs [50–52]. Cheng et al. [53] presented an alternative approach called Selective Organ Targeting (SORT), where they showed that by the inclusion of an additional cationic or anionic lipid (i.e.*,* SORT molecule), activity profiles of mRNA LNPs could be shifted from the liver to the lungs and spleen. Although the authors could clearly demonstrate organ-specific mRNA expression, it remains to be elucidated whether this can be the result of different cellular uptake patterns for each of the investigated mRNA LNPs. Finally, mRNA LNPs are also applicable to other administration routes, including the direct injection of mRNA therapeutics in tumor lesions [54, 55]. Of note, mRNA vaccines using LNP technologies for intramuscular administration are set to become the first vaccines in the fight against COVID-19 [56, 57]. Together, LNPs have strongly catalyzed the clinical applicability of mRNA therapeutics and based on their design flexibility, opened a wide range of new therapeutic opportunities.

### The applications of mRNA therapeutics for cancer immunotherapy beyond tumor antigen vaccination

#### mRNA for delivery of mAbs

**Fig. 2:**
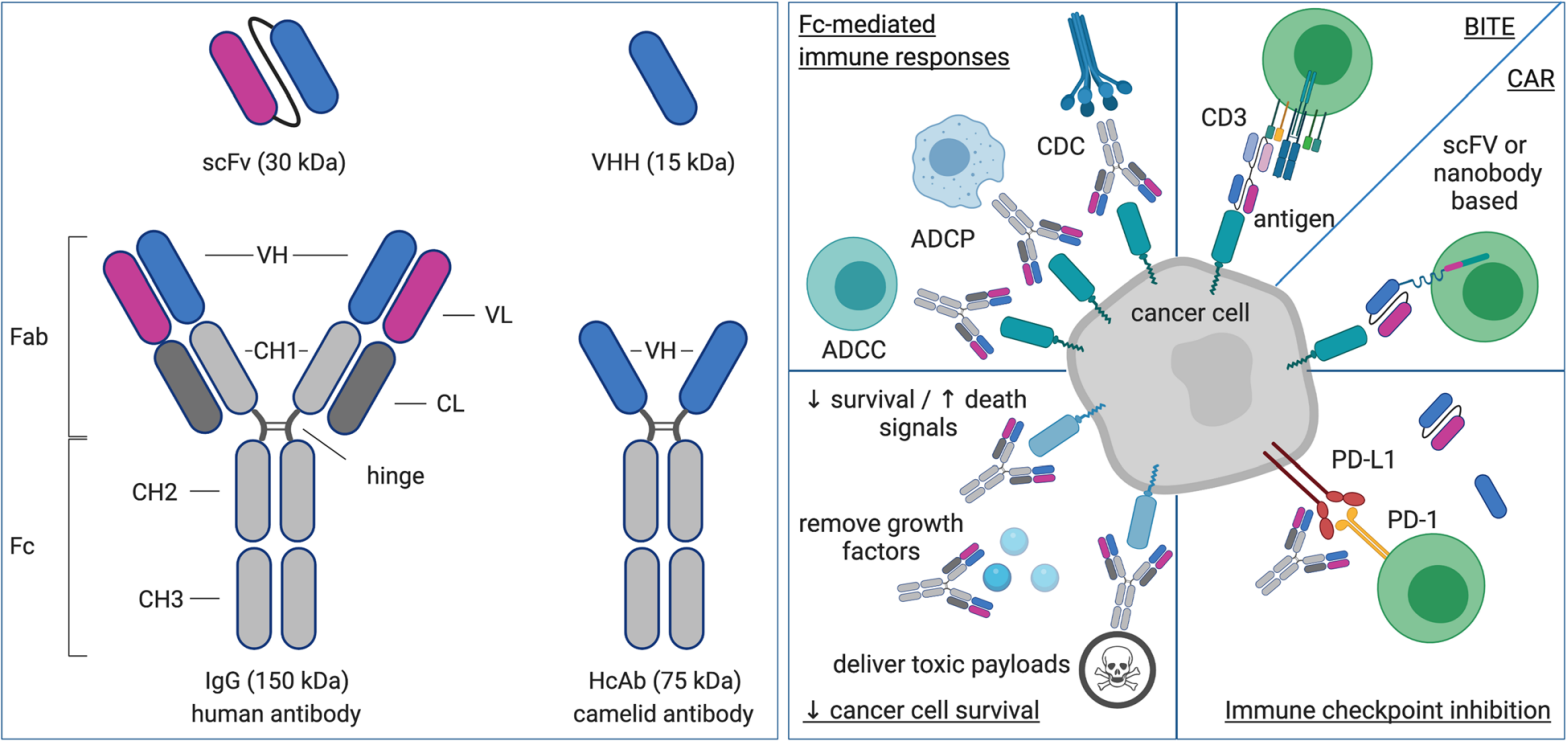
The structure and application of antibody (fragments) for cancer therapy. Left box: IgG antibodies (150 kDa) as found in humans consist of a Fab and Fc fragment, which are linked through a hinge region. The Fc fragment consists of constant regions from the heavy chain (CH), while the Fab fragment consists of constant and variable domains of the heavy and light chain (CH, VH and CL, VL respectively). Heavy chain only antibodies (HcAbs, 75 kDa) as found in camelids lack light chains. Examples of antibody fragments are single chain variable fragments (scFvs, 30 kDa) and heavy chain only VH domains (VHHs, nanobodies, 15 kDa). Right box: Antibodies are exploited to induce Fc-mediated anti-tumor immunity through activation of complement factors (CDC), macrophages (ADCP) or NK cells (ADCC). Antibodies and antibody fragments are also used to activate T cells with cytolytic activity. Bispecific T cell engagers (BiTEs) form a bridge between cancer cells and T cells. Chimeric antigen receptors (CARs) allow T cells to recognize and kill cancer cells independent of antigen presentation in human leukocyte antigens (HLAs). Blockade of inhibitory immune checkpoints, such as that consisting of programmed death-1 (PD-1) and its ligand (PD-L1), releases the brake on T cell activity. Antibodies and antibody drug conjugates are exploited to directly act on cancer cells, depriving them from survival signals or growth factors, activating cell death pathways or delivering toxic payloads. Abbreviations: ADCC: antibody-dependent cellular cytotoxicity; ADCP: antibody-dependent cell phagocytosis; BiTE: bispecific T cell engager; CAR: chimeric antigen receptor; CDC: complement-dependent cytotoxicity; CH: constant domain of the heavy chain; CL: constant domain of the light chain; Fab: fragment, antigen binding; Fc: fragment crystallizable region; HcAb: heavy chain only antibody; HLA: human leukocyte antigen; PD-1: programmed death-1; PD-L1: programmed death-ligand 1; VH: variable domain of the heavy chain; VHH: variable domain of the heavy chain only antibody; VL: variable domain of the light chain

Recombinant, full-sized mAbs and antibody fragments, such as single chain variable fragments (scFv) and heavy chain only VH domains (VHHs, nanobodies), that target tumor antigens, tumor stromal factors, immune cells or immune pathways, have been studied as therapeutics in the field of immune-oncology (Fig. 2) [58, 59]. Moreover, antibody fragments have been used as building blocks for bispecific antibody formats and chimeric antigen receptors (CARs), a component in adoptive cell therapy, thereby making antibody fragments an invaluable asset in the immuno-oncology arsenal.

Many challenges associated with the use of mAbs and antibody fragments became apparent with the advancement of this field, among which cost-efficient production of these proteins and ensuring sufficiently high levels at the site of action, two challenges for which it is contended that IVT mRNA can provide a solution [60, 61]. mRNA is capable of encoding any protein. Therefore, it is possible to encode full-size mAbs, antibody fragments or any variants designed with these antibody fragments. The delivery of this mRNA to cells and the subsequent production of these proteins by the cells ensures proper assembly and post-translational modification, which is particularly critical for the function of full-size mAbs [62]. The mRNA molecule and its mode of delivery can be tweaked to ensure prolonged protein expression at the site of interest, for instance the TME, implying that mAb levels can be sustained locally and over a period of time. This holds the promise of increased efficacy and decreased toxicity. Research to evaluate mRNA as an efficient vector for delivery of (bispecific) antibodies has been performed in view of activating anti-tumor immune responses. The choice for focusing on these approaches is obvious, as jumpstarting the anti-tumor immune response could be sufficient to set off a cascade of immune activities that cooperate to eradicate cancer cells, providing long-lasting benefit.

We refer to excellent reviews covering (1) the deprivation of cancer cells from nutrients and oxygen through the use of mAbs that target vascular growth factors or stromal factors, ultimately resulting in cancer cell death [63] as well as (2) the targeting of receptors on cancer cells to block essential growth signals [64], to deliver cell death-inducing signals [65], or to simply act as anchoring points for delivery of toxic payloads [66]. In this section, we will focus on how mRNA encoding antibodies and their derivatives has been exploited to fight cancer and how the mRNA-encoded antigen-binding moiety activates anti-tumor immunity making use of different strategies.

One strategy that relies on the activation of anti-tumor immunity involves leveraging of effector functions of the Fc region of cancer-specific mAbs to initiate complement-dependent cytotoxicity (CDC), antibody-dependent cellular phagocytosis (ADCP) or antibody-dependent cellular cytotoxicity (ADCC) [67]. Rituximab is a clinically approved IgG1 mAb that targets CD20 and induces CDC and to lesser extent ADCC [68]. This mAb has been encoded in mRNA, an approach that has shown benefit in a preclinical B cell lymphoma model. More specifically, Thran et al. [69] encoded the codon-optimized sequence of the rituximab heavy and light chain on two separate plasmids for production of mRNA. The mRNA was produced with unmodified nucleotides, purified using HPLC, admixed at equimolar ratios and formulated in LNP for in situ delivery to hepatocytes. Mice bearing CD20^+^ Raji cells were treated twice per week with escalating doses of the rituximab mRNA-LNP, showing dose-dependent tumor control. Notably, a significant number of mice showed tumor rejection at the 50 μg rituximab mRNA-LNP dose, while mice treated with 200 μg rituximab (control group) showed a worse outcome.

Second, particular attention should be devoted towards the use of bispecific antibodies that simultaneously bind cancer- and T cell-specific surface proteins, thereby redirecting and activating T cells to kill cancer cells [70]. To that end, a scFv from an antibody targeting a cell surface molecule on T cells (often CD3ε) is linked through a short flexible linker to a scFv from an antibody targeting an antigen on the surface of cancer cells. These are commonly referred to as bispecific T cell engagers or BiTEs [71]. The BiTEs that were so far encoded in IVT mRNA targeted CD3 in combination with claudin 6 (CLDN6) or epithelial cell adhesion molecule (EpCAM) [46]. The IVT mRNA used to encode these BiTEs was improved in terms of stability and translational efficacy through modifications to the UTRs, cap and poly(A) tail. The transfection reagent TransIT® was used to encapsulate the BiTE mRNA, and to mediate its uptake and expression in the liver upon intravenous injection. Using modified nucleotides during the production of mRNA was required to obtain high BiTE plasma levels. Notably, BiTE plasma levels were still detectable at 72 h, at which time recombinant BiTE proteins, the control, were no longer detectable in the plasma. Picomolar concentrations of the mRNA-encoded BiTEs was sufficient to mediate cell lysis in an ex vivo cytotoxicity assay. In vivo*,* both CLDN6xCD3 and EpCAMxCD3 BiTEs encoded in mRNA were used to treat ovarian carcinoma (OV-90) and human peripheral blood mononuclear cell transplanted NSG mice. The treatment was performed over a period of 3 weeks and consisted of 3 μg of BiTE mRNA per week. For comparison, a cohort of mice was treated for 3 weeks with three to four injections per week of 200 μg/kg of recombinant BiTE proteins. Tumor control was reached in all mice.

Finally, antibodies that act on immune checkpoints, pathways installed to stimulate or inhibit immune activation, have revolutionized standard-of-care for several cancer indications. In this regard, mAbs blocking the inhibitory immune checkpoint consisting of cytotoxic T lymphocyte-associated antigen-4 (CTLA-4), programmed death-1 (PD-1) and its ligand PD-L1, are considered game changers in the field of oncology [72, 73]. As a more recent development, nanobodies that bind and block CTLA-4 or PD-L1 have shown potential in view of immune activation at the priming as well as the effector stage of T cells [74–77]. However, as these pathways are critical regulators of T cell activation, attention should be paid towards careful regulation of amongst others optimal doses and administration schedules to avoid immune related auto-reactivity and toxicity [78]. To circumvent these safety issues, the use of mRNA encoding immune modulators can be considered as a promising strategy. Although, Pruitt et al. described local modulation of immune checkpoints at the TME, this was achieved by ex vivo transfection of DCs with mRNA encoding the H and L chains of anti-CTLA-4 and anti-GITR mAbs [79]. Based on this technology, a clinical study for patients with metastatic melanoma was initiated (NCT01216436). Thus far, studies focusing on the in vivo manipulation of immune modulators using mRNA have not yet been reported, although preclinical studies on this topic are ongoing.

All together, these studies are a first testimony of the potential of mRNA-mediated delivery of antibodies and antibody derivatives, and warrant further research into this technology platform for systemic or local production of these proteins in a transient fashion, yet achieving a more interesting pharmacokinetic profile when compared to delivery of recombinant proteins.

#### mRNA for induction of cell death in cancerous cells

**Fig. 3:**
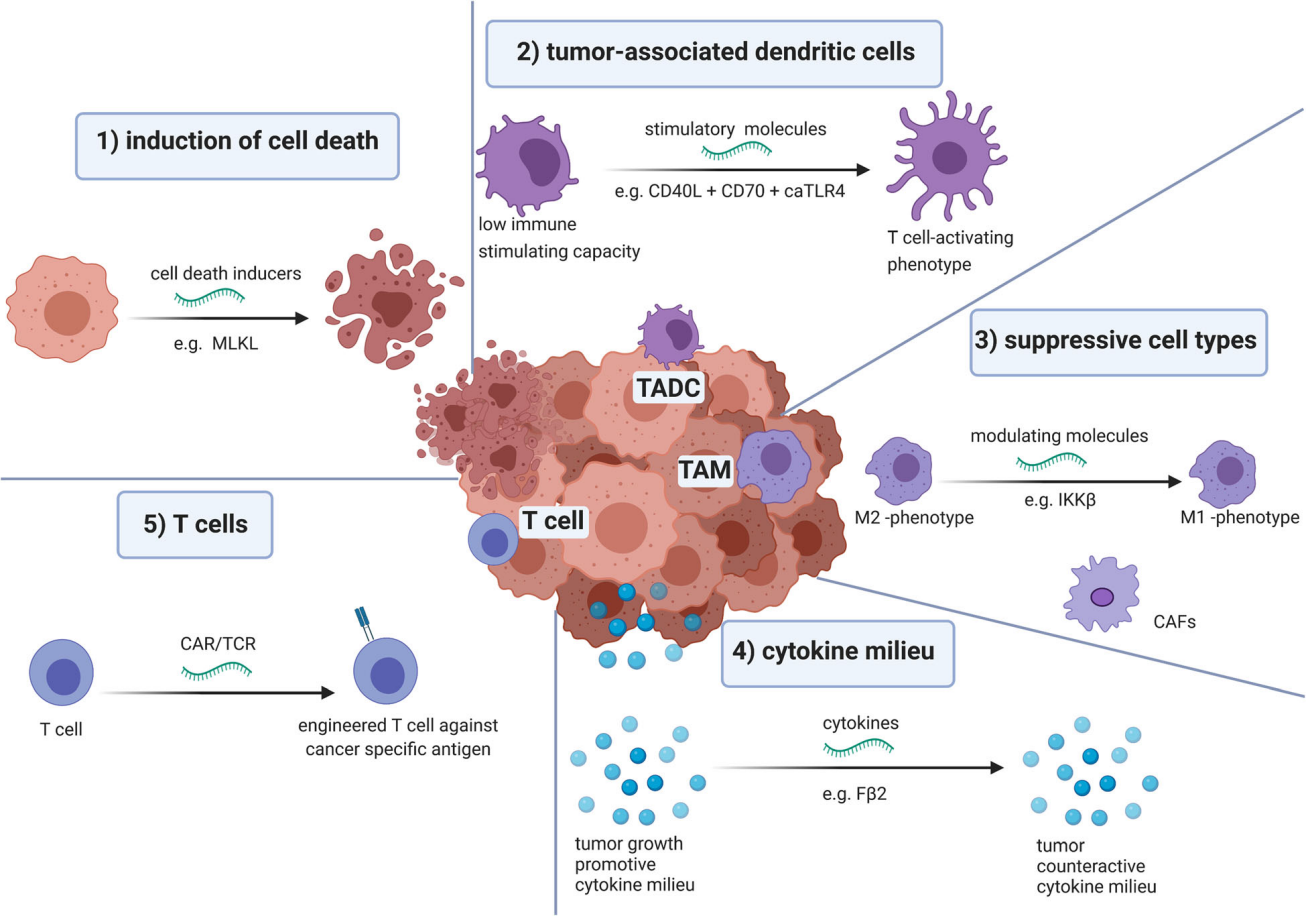
The in vivo application of mRNA to modulate the tumor microenvironment. (1) Use of mRNA for induction of cell death in diseased cells *e.g*. mRNA encoding MLKL enforce cells to die in an immunogenic way [80] (2) Use of mRNA to modulate tumor-associated dendritic cells (TADC) *e.g*. mRNA encoding for CD40L, CD70 and caTLR4 (TriMix) can be combined to stimulate immature TADC to mature TADC [81–84]. (3) Use of mRNA to modulate suppressive cell types *e.g*. mRNA encoding IKKβ is used to genetic reprogram tumor-associated macrophages [85]. (4) The use of mRNA to modulate the cytokine milieu *e.g*. mRNA encoding IFN-β fused to the ectodomain of the TGF-β receptor II (Fβ_2_) [86]. (5) Use of mRNA for generation of cancer-specific T cells. mRNA can be used to genetic engineer T cells to express cancer-specific T cell receptors or chimeric antigen receptor. Abbreviations: MLKL: Mixed lineage kinase domain like pseudokinase; CD40L: Cluster of differentiation 40 ligand; CD70 Cluster of differentiation 70; caTLR4: constitutive active toll like receptor 4; M2: macrophage type 2 phenotype; M1: macrophage type 1 phenotype; IKKβ: Inhibitor of nuclear factor kappa-β kinase; MDSC: Myeloid-derived suppressor cell; CAF: Cancer-associated fibroblast; Fβ_2_: IFN-β fused to the ectodomain of the TGF-β receptor II; TCR: T cell receptor; CAR: chimeric antigen receptor

mRNA therapeutics can also be used to force diseased cells to synthesize a toxic intracellular protein, causing cells to self-destruct (Fig. 3, panel 1). For this type of approach, it is of utmost importance to express the toxic protein exclusively in the target cells. Unfortunately, current nanoparticle-based mRNA delivery methods exhibit a high propensity for expression in the liver upon systemic administration. To circumvent this, Jain and colleagues showed that the inclusion of microRNA target sites in therapeutic mRNAs encoding apoptotic proteins, caspase or PUMA, can prevent their expression in healthy hepatocytes while triggering apoptosis in hepatocellular carcinoma cells [87]. Such miRNA-mediated elimination of liver toxicity could be very effective for systemic administration of modified mRNAs.

Next to this approach, Van Hoecke and colleagues evaluated intratumoral injection of hypo-inflammatory mRNA encoding the key execution protein in the necroptosis pathway, MLKL, immediately followed by electroporation of the tumor [80]. Moreover, hypo-inflammatory mRNA was manufactured for delivery of tBID [88], the truncated Bcl2-like inducer of cell death, which induces apoptosis. In vitro transfection of B16 (melanoma) and CT26 (colon carcinoma) tumor cells with MLKL or tBID was achieved using Lipofectamine™ as a transfection agent, while in vivo delivery of the mRNA was achieved through electroporation. Both tBID and MLKL mRNA induced significant cell death in vitro and delayed tumor growth in vivo*.* In these experiments, mRNA encoding MLKL showed the best therapeutic index. It was shown that intratumoral delivery of MLKL mRNA stimulates anti-tumor immunity against neo-epitopes, a process that was dependent on CD8α^+^ DCs, which are proficient in antigen cross-presentation. Moreover, it was shown that type I interferon (IFN) was key in the immune activation [80].

#### The use of mRNA to modulate tumor-associated dendritic cells

Several DC subtypes have been identified in the TME of different murine and human cancers. Commonly, these are referred to as tumor-associated DCs (TADCs). Although the function of TADCs is partly lineage-specific, where specific subsets such as conventional DC1 seem hardwired for efficient cross-presentation of tumor antigens, a large body of evidence points to corruption of immunogenic DC functions by TME-related factors. TADCs are rendered defective in their immune activating capacity among others by hypoxia, accumulation of adenosine and lactate, and a number of cytokines, including vascular endothelial growth factor (VEGF), interleukin-10 (IL-10), and prostaglandin E2 (PGE2) [89–93]. Nonetheless, it was shown that TADCs can acquire tumor antigens and when lifted from the TME can activate tumor antigen-specific T cell responses [92].

Therefore, reprogramming TADCs (Fig. 3, panel 2), to acquire a T cell-activating phenotype, has been studied using an mRNA-based approach, in particular by intratumoral injection of TriMix mRNA [94]. TriMix is an mRNA-cocktail composed of three mRNA molecules encoding CD40L, CD70 and a constitutive active form of TLR4 (caTLR4) and could be considered as a new standard for DC activation [81–84]. Local administration of so-called “naked” mRNA results in its selective uptake by and expression in cross-presenting CD8α^+^ DCs. As a consequence, injection of TriMix mRNA enables reprogramming of CD8α^+^ TADCs, as shown by the acquisition of a mature phenotype and the ability to migrate to lymph nodes and activate T cells. By injecting tumor-bearing mice with TriMix mRNA, a delay in tumor growth was obtained without the need to co-deliver defined tumor antigens. This possibility to exploit the tumor’s own antigenic repertoire by TriMix administration was shown in models of primary as well as disseminated tumors [94]. Currently a clinical trial to study the safety and immunomodulatory effect upon intratumoral delivery of TriMix mRNA in patients with early breast cancer is ongoing (NCT03788083).

Similar to this study Haabeth et al. [95] used charge-altering releasable transporters (CARTs) to deliver mRNA encoding co-stimulatory molecules to TADCs in a two-sided A20 model, representing disseminated cancer. Notably, CARTs enabled transfection of T cells and tumor-associated macrophages (TAMs) as well. Delivery of CD70 mRNA only marginally affected tumor growth in both the treated and non-treated tumor. In contrast, CART-mediated delivery of OX40L mRNA induced regression in 100% of treated tumors, while a delay in growth was observed in the non-treated tumors. Similar results were obtained upon delivery of CARTs loaded with mRNA that encoded the co-stimulatory molecules CD80 and CD86. It was shown that transfected TADCs migrated to lymph nodes, suggesting a similar mode of action as described by Van Lint *et al* [94]*.* Nonetheless, it cannot be excluded that T cells and TAMs contribute to this therapeutic effect. Transfected T cells were also detected in lymph nodes, at least suggesting that these T cells could exert effector functions at distal sites.

#### The use of mRNA to modulate suppressive cell types in the tumor microenvironment

The tumor forms a dense network of both malignant and non-malignant cells. Several immune cells present in the TME are key regulators in the immunosuppressive milieu and as such further contribute to the tumor-promoting angiogenesis and formation of metastases [96, 97]. Of these, cancer-associated fibroblasts (CAFs) and TAMs can be considered as the most abundant non-neoplastic cells found in the TME (Fig. 3, panel 3).

TAMs show the remarkable potential to respond to environmental stimuli and many studies have shown that modulation of TAMs can elicit anti-tumor T cell responses [98–101]. Genetic reprogramming of TAMs using IVT mRNA has recently been described [102]. Intratumoral delivery of mRNA encoding costimulatory and immune modulating factors by means of the CART-nanoparticle technology, not only induced local modulation of TADC as described in the previous paragraph but additionally showed to be able to transfect TAMs as well [95]. In line with this, Seif et al. described the preferential transfection of type 2 TAMs upon recombinant yeast delivery of biosynthesized mRNA. As such, selective delivery of mRNA encoding the pro-inflammatory regulators Myd88 and TNF to pro-tumorigenic type 2 TAMs resulted in upregulation of pro-inflammatory and cytotoxic cytokines and suggested re-education towards TAMs with a pro-immunogenic profile [103]. On top, Interferon Regulatory Factor 5 (IRF5) and IKKβ, a kinase that induces phosphorylation and activation of IRF5 were shown as ideal master regulators of TAM polarization to finally imprint TAMs with a potent pro-inflammatory phenotype [85, 104]. Delivery of a targeted nanocarrier formulated with IVT mRNA encoding IRF5 and IKKβ, induced reversion of the suppressive state of TAMs from a tumor-supporting phenotype towards a phenotype that induced anti-tumor immunity and promoted tumor suppression [102]. Showing safety and efficacy in various preclinical mouse tumor models (including ovarian cancer, melanoma and glioblastoma), intraperitoneal administration of this technology to treat ovarian cancer patients as a first clinical translation of this therapy is envisaged (Fed Hutchinson Cancer Research Center).

In several cancers, intrinsic resistance to immunotherapy is upheld by a connective tissue “shield” consisting of dense extracellular matrix which is maintained by CAFs. In addition, CAFs secrete a myriad of immunosuppressive factors. Selective targeting of CAFs, or more specifically the CAF-specific fibroblast activating protein (FAP), is capable of precipitating tumoral collapse in preclinical tumor models [105, 106]. Although this has spurred interest in the development of CAF-targeted vaccine or CAR T-cell approaches, the potential of an mRNA-based approach in this setting warrants research efforts as well [107]. The feasibility of such an approach is highlighted by several studies that used LNPs for delivery of plasmid DNA to CAFs, showing that delivery of cell death inducing genes [108] or immunotherapeutic agents [109, 110] is a valid approach.

#### The use of mRNA to modulate the cytokine milieu in the tumor microenvironment

The cytokine milieu in tumors is determined by production of various cytokines by immune and stromal cells (*e.g*.*,* fibroblasts, endothelial cells) [111]. It is the balance of pro- and anti-inflammatory cytokines, their relative concentration in combination with the cell composition in the TME, hence type of cells that can be affected by these cytokines, that determine the net effect of the cytokine milieu [112]. This net effect can be categorized as promoting versus countering tumor development and dissemination. To date, several studies have exploited mRNA as a vehicle to modulate the cytokine milieu (Fig. 3, panel 4) [54, 55, 86, 113].

Intratumoral delivery of mRNA encoding a fusokine called Fβ^2^, which consisted of IFN-β fused to the ectodomain of the TGF-β receptor II [86], was evaluated to mimic the observations that were made earlier using a combination of IFN-β and a TGF-β signaling antagonist [114]. The idea behind this approach is dual: IFN-β can increase immune reactivity, while the ectodomain of the TGF-β receptor II can reduce TGF-β mediated immunosuppression. The mRNA-encoded fusokine reduced the immunosuppressive capacity of MDSCs, while it enhanced the T cell stimulatory activity of DCs. Tumor cell proliferation was affected by Fβ^2^ as well, although it was observed that surviving tumor cells expressed high levels of PD-L1. In situ delivery of Fβ^2^ resulted in a delay in tumor growth, which could be further enhanced through additional blockade of PD-1/PD-L1 interactions [86]. The Fβ^2^ mRNA was delivered in its “naked” form to tumors and as such depended on TADCs to serve as a kind of factories to produce and secrete the fusokine.

Several pharmaceutical formulations have been studied for delivery of mRNA that encodes for cytokines to cells residing in the TME [54, 55, 95]. Cytokines that have been studied in this context are IL-12, IL-23, IL-36γ and IFN-γ. Most of these cytokines have a narrow therapeutic window with a poor safety profile when administered systemically [115, 116]. Their local delivery using mRNA formulated in LNPs represents a safe approach to leverage these cytokines anti-tumor effects. To initiate responses to foreign pathogens, IL-36γ is a classic alarm signal, whereas IL-23 centrally coordinates immune responses to danger signals. The intratumoral delivery of mRNA encoding these two cytokines induced a robust anti-cancer response in different tumor models. On top, the addition of mRNA encoding OX40 ligand (OX40L), a T cell costimulatory cytokine, further increased the response rates. Mechanistically, this treatment is dependent on Batf3-dependent cross-presenting CD8α^+^ DCs and cytotoxic CD8^+^ T cells. The combination of the IL-23/IL-36γ/OX40L triplet mRNA mixture with checkpoint blockade was efficient in models otherwise resistant to systemic immune checkpoint inhibition [54]. Similarly, the use of mRNA encoding IFN-γ or IL-12 either alone or in combination with OX40L mRNA has been tested in a model of disseminated cancer [95]. The mRNA was delivered using CARTs to one tumor, as such modifying and immediately acting on multiple cells in the TME. This study showed that delivery of IFN-γ mRNA delayed overall tumor growth, however, to a lesser extent as delivery of IL-12 mRNA. Co-delivery of OX40L mRNA enhanced the effect of both cytokines, resulting in a further delay of tumor growth in case IFN-γ and OX40L mRNA was delivered and even cure of several mice when IL-12 and OX40L mRNA was delivered [95]. This outcome is not unexpected as IL-12 is an important cytokine, known to guide the differentiation of T helper 1 (Th1) cells as well as enhancing the cytotoxic activity of NK cells, NKT cells and CTLs [117]. Hewitt et al. showed that a single intratumoral injection of LNP-formulated IL-12 mRNA induced strong CTL-dependent tumor regression, systemic effects on distant tumor sites and long-term immunity. The induced effects were accompanied with transformation of the Th1 TME [55]. These preclinical data are at the basis of a phase I clinical trial in which patients with solid tumors receive IL-12 mRNA intratumorally in combination with the PD-L1 blocking antibody darvulumab (NCT03946800). Also, Malkova et al. described local delivery of mRNA encoding single chain IL-12, albeit in combination with IFN-α, granulocyte macrophage-colony stimulating factor (GM-CSF) and interleukin-15 (IL-15). This mRNA-based cytokine cocktail was delivered either alone or in combination with anti-PD-1 treatment against various murine tumor types. Preliminary results showed significant anti-tumor responses when the mRNA-based cytokine mix was delivered alone, which could be further improved by combination with anti-PD-1 treatment [113]. A first-in-human dose escalation and expansion study was initiated in patients with advanced solid tumors (NCT03871348).

#### mRNA for generation of cancer-specific T cells

Adoptive T cell therapy is a catch-all term that covers therapeutic approaches in which enriched or engineered autologous cancer-specific T cells are administered. Pioneering work was performed in the ‘80s by Steven A. Rosenbergh, who first described cancer regression after the infusion of isolated, autologous tumor-infiltrating lymphocytes (TILs) in combination with high doses IL-2 in patients with metastatic melanoma [112]. Unfortunately, translation of this concept to other tumor types was found difficult, requiring new ways to generate cancer-reactive lymphocytes. This prompted genetic engineering of T cells to express cancer-specific T cell receptors (TCRs) or CARs (Fig. 3, panel 5) [4].

Adoptive T cell transfer classically entails ex vivo modification of isolated autologous cells for which a plethora of techniques and compounds for T cell engineering have been evaluated, including mRNA*.* The most prominent modification to T cells is the induction of CAR or TCR expression, which is mainly achieved by the use of retro- or lentiviral transduction, which unfortunately entails a risk of insertional mutagenesis. In this light, electroporation of CAS9 mRNA into human T cells was used as an alternative for randomly integrating viral vectors, as it allowed directed integration of a CD19-specific CAR to the T cell receptor α constant (TRAC) locus. This resulted in uniform CAR expression as well as enhanced T cell potency [118]. Also, mRNA encoding CARs or TCRs has shown merit: inducing temporary expression of CARs or TCRs could avoid the occurrence of the cytokine release syndrome, an adverse event related to permanent T cell activity [119–122]. Although reports indeed describe clear therapeutic benefits of mRNA-CAR T cell therapies, the other side of the coin is the induction of human anti-mouse antibodies (HAMA) due to the repeated dosing of mRNA-modified CAR T cells, which were reported to result in IgE-mediated anaphylactic shock [123]. Furthermore, mRNA also showed potential to improve the functionality of therapeutic T cells, *e.g*., by transfection with mRNA encoding (membrane-bound) cytokines [124, 125]. The use of mRNA for modification of T cells and its preclinical efficacy as well as evaluation in a clinical setting is extensively reviewed elsewhere [126].

It is evident that in these applications, the main goal is to achieve high, but temporary, gene expression. This provides a clear rationale for the use of minimally immunogenic mRNA with a high translation efficiency. Indeed, murine T cells electroporated with CAR-encoding mRNA, were shown to exhibit a drastically reduced up-regulation of checkpoint molecules (PD-1 and LAG-3) when chemically modified 1mΨ mRNA and/or mRNA that was additionally purified to remove dsRNA was used, as compared to their unmodified and unpurified counterparts. This equipped the immunosilent mRNA-transfected T cells with an improved killing efficacy that lasted even after the CAR expression by the cells was already lost [32].

Despite successes obtained with T cell therapies in hematological malignancies and the recent FDA approval of the third CAR T cell therapeutic [127], their complex manufacturing and difficulties in upscaling remain largely unaddressed. Unfortunately, directly modifying T cells in vivo, as compared to APCs, is an arduous task as T cells are notoriously hard to transfect. A few promising reports were published on the in vivo engineering of CAR T cells via administration of lentiviral vectors [128, 129] or DNA-nanoparticles [130]. For mRNA, merely a single study reports on the use of binary mixtures of CARTs for transfection of T cells, albeit with limited efficiency and selectivity: only 1.5% of splenic CD8^+^ T cells were transfected after intravenous administration of the mRNA-CARTs, while at least 10-fold higher transfection rates were observed in macrophages, DCs and B cells [131].

### Bringing mRNA-based immunomodulation to oncological practice: current challenges and future perspectives

For every new candidate anti-cancer therapy, a successful journey from promising experimental concept to successful implementation in patients entails major risks which are situated at 3 levels: manufacturing, safety and demonstration of clinical efficacy. In the case of mRNA, the capacity to manufacture a GMP-compliant nanoparticle-based product at (very) large scale has recently been demonstrated in the context of Covid-19 vaccine development. The experience that is being acquired with respect to industrial-scale production, storage and distribution will undoubtably benefit the field of mRNA-based cancer therapeutics as well.

As for safety, awareness of the regulatory framework where mRNA-based therapeutics are situated today is important. Despite the limited persistence of mRNA in vivo and the virtually non-existent risk for genomic integration or insertional mutagenesis, both EMA and FDA consider mRNA-based medicines as *bona fide* gene therapy. This perception may change however considering the way regulatory authorities are fast-tracking approval of mRNA-based vaccines against SARS-Cov2 for deployment at massive scale. Still, transition to first-in-human studies require prior rigorous documentation of pharmacokinetics, pharmacodynamics, biodistribution and shedding of the product. Because mRNA-based immunomodulators are primarily targeted at disseminated cancers, these agents will most often be administered via systemic route. Specifically targeting the injected dose to the microenvironment of tumoral sites rather than normal tissue in a given organ or in multiple affected organs is a considerable clinical challenge. Preclinical strategies allowing to reduce hepatic accumulation (and resulting toxicity), or even favor distribution to spleen or lung are still too crude with that respect [53, 87]. More cancer-selective targeting approaches may exploit the extreme metabolic environment inside aggressively developing tumor beds, *i.e*. the profound hypoxia and lactate-induced acidic *p*H. The physicochemical properties of LNPs could be fine-tuned to favor selective trapping and accumulation in the acidic intratumoral environment. Alternatively, incorporation of hypoxia-activated prodrugs in the nanoparticle formulation may yield novel ways to target the mRNA cargo to fast growing tumors where oxygen levels are extremely low. A different strategy may use nanoparticle carriers functionalized with molecules allowing targeting to tumor-specific vascular markers [132]. For instance, RGD-peptides or anti-CD105 antibodies can endow nanomaterials with specificity to resp. αvβ3 integrin or endoglin, both of which are highly enriched on the intratumoral endothelial surface.

As the lethality of many cancers results from the development of cerebral metastases which are typically refractory to many existing therapies, designing mRNA carrier formulations able to cross the blood-brain barrier could be a real game-changer. A successful proof-of-concept of this approach was recently described, making use of LNPs doped with neuro-transmitter precursor molecules, and containing an active cargo -*in casu* an antisense RNA molecule [133].

Whichever formulation used for an mRNA-based cancer therapeutic, clinical development will in most cases involve a combination regimen, typically on top of existing standard-of-care cancer therapy, be it radiotherapy, chemotherapy, oncogene-targeted therapy, immune checkpoint blockade or adoptive T cell therapy. Combination trials will dominate the clinical trial landscape in immuno-oncology for years to come, with the large majority evaluating an investigational drug on top of an immune checkpoint inhibitor backbone (reviewed in Tang et al. [134]). The spectacular and seemingly anarchic proliferation of immuno-oncology combination trials calls for a more rationalized approach, where the combination partner will have to be selected based on a profiling of the dominant immunosuppressive mechanism for a given tumor or patient. Early steps towards this “precision immuno-oncology” paradigm are already being taken, especially in tumor entities with a high occurrence of primary or acquired resistance to standard-of-care immunotherapeutics [135, 136]. With its inherent versatility, mRNA-based immunomodulation seems extremely well suited to be integrated in this approach.

## Conclusion

IVT mRNA has tremendous potential to become the basis of a disruptive technology in oncology. This is due to the promise mRNA therapeutics hold as effective, safe and affordable strategy. Moreover, the growing knowledge of formulations that allow protection of the mRNA as well as selective delivery of the mRNA to an organ or cell of interest has further boomed the field of mRNA therapeutics. Although tremendous progress in research and the promising potential, the latter still poses a challenge that needs more in-depth investigation on how LNP formulations can specifically deliver mRNA to the desired target. While some cell-specific LNPs have been developed, *e.g*. clinically approved “Onpattro” formulation, it is still a large unknown which specific immune cell subsets are targeted by playing with LNP compositions.

The first field of entry for mRNA therapeutics was the cancer vaccination field. The development and application of mRNA therapeutics for treatment of cancer has boomed in the past years. In general, mRNA-based cancer vaccination can be divided in two approaches. The most obvious one is the immunization of patients with mRNA encoding tumor antigens. As mRNA therapeutics have a flexible production process, this patient immunization can be done on a personalized level. The second approach explores the use of mRNA to reshape the TME by delivering mRNA encoding stimulatory molecules, blockade of inhibitory molecules and immune checkpoint blockade to restore immunological fitness at the tumor site. The immunosuppressive TME is a major obstacle in cancer immunotherapy. Thanks to a growing insight into the suppressive mechanism of the TME, possible ways to block tumor escape are currently under investigation, including the delivery of cytokines, decoy receptors or other secreted immune modulatory proteins.

Today, much effort is put into the use of immune checkpoint inhibitors. However, immune checkpoint blockade fixes only one late step in the chain leading to T cell induced tumor destruction. To achieve complete and safe eradication of tumor cells, cancer immunotherapy should focus on combined therapies. This is key to outsmart tumor escape mechanisms and (re-)induce an equilibrium state of cancer immunoediting. As described in this review, many strategies using mRNA as a vector for in situ delivery of therapeutic proteins to restore immunological fitness at the tumor site.

Altogether, the use of mRNA-based strategies has evolved the therapeutic landscape at high speed. In this fast-evolving world of cancer therapy, future combinations of mRNA-based immunotherapies should be matter of investigation.

## Data Availability

Not applicable.

## Abbreviations

1mΨ: N1-methyl-pseudouridine
ADCC: Antibody-dependent cellular cytotoxicity
ADCP: Antibody-dependent cell phagocytosis
APC: Antigen-presenting cell
BiTE: Bispecific T cell engager
CAR: Chimeric antigen receptor
CART: Charge-altering releasable transporters
CDC: Complement-dependent cytotoxicity
CH: Constant domain of the heavy chain
CL: Constant domain of the light chain
CLDN6: Claudin 6
CTL: Cytotoxic T Lymphocyte
DAMP: Damage-associated molecular patterns
DC: Dendritic cells
dsRNA: double-stranded RNA
EpCAM: Epithelial cell adhesion molecule
Fab: Fragment, antigen binding
Fc: Fragment crystallizable region
HA: Hemagglutinin
HAMA: Human anti-mouse antibodies
HcAb: Heavy chain only antibody
HLA: Human leukocyte antigen
HPLC: High-performance liquid chromatography
IC50: Half maximal inhibitory concentration
IVT: In vitro transcribed
LNP: Lipid-based nanoparticle
mAb: monoclonal antibody
MDSC: Myeloid-derived suppressor cell
MDA: Melanoma-differentiation-associated
NK: Natural killer
ORF: Open reading frame
PD-1: Programmed death-1
PD-L1: Programmed death-ligand 1
PEG: Polyethylene glycol
PRR: Pattern recognition receptor
RIG-I: Retinoic-acid-inducible protein 1
scFv: single chain variable fragments
siRNA: small interfering RNA
TAA: Tumor-associated antigens
TAM: Tumor-associated macrophage
TCR: T cell receptor
TIL: Tumor-infiltrating lymphocytes
TLR: Toll-like receptor
TME: Tumor microenvironment
TRAC: T cell receptor α constant
Treg: Regulatory T cell
UTR: Untranslated region
VH: Variable domain of the heavy chain
VHH: Variable domain of the heavy chain only antibody or nanobody
VL: Variable domain of the light chain
